# A longitudinal experiment demonstrates that honey bee colonies managed organically are as healthy and productive as those managed conventionally

**DOI:** 10.1038/s41598-023-32824-w

**Published:** 2023-04-13

**Authors:** Robyn M. Underwood, Brooke L. Lawrence, Nash E. Turley, Lizzette D. Cambron-Kopco, Parry M. Kietzman, Brenna E. Traver, Margarita M. López-Uribe

**Affiliations:** 1 Department of Entomology, The Pennsylvania State University, University Park, PA USA; 2 School of Plant and Environmental Sciences, Virginia Tech, Blacksburg, VA USA; 3 Department of Biology, Penn State Schuylkill, Schuylkill Haven, PA USA

**Keywords:** Agroecology, Population dynamics, Ecology, Zoology, Diseases

## Abstract

Honey bee colony management is critical to mitigating the negative effects of biotic and abiotic stressors. However, there is significant variation in the practices implemented by beekeepers, which results in varying management systems. This longitudinal study incorporated a systems approach to experimentally test the role of three representative beekeeping management systems (conventional, organic, and chemical-free) on the health and productivity of stationary honey-producing colonies over 3 years. We found that the survival rates for colonies in the conventional and organic management systems were equivalent, but around 2.8 times greater than the survival under chemical-free management. Honey production was also similar, with 102% and 119% more honey produced in conventional and organic management systems, respectively, than in the chemical-free management system. We also report significant differences in biomarkers of health including pathogen levels (DWV, IAPV, *Vairimorpha apis*, *Vairimorpha ceranae*) and gene expression (*def-1*, *hym*, *nkd*, *vg*). Our results experimentally demonstrate that beekeeping management practices are key drivers of survival and productivity of managed honey bee colonies. More importantly, we found that the organic management system—which uses organic-approved chemicals for mite control—supports healthy and productive colonies, and can be incorporated as a sustainable approach for stationary honey-producing beekeeping operations.

## Introduction

Colonies of the Western honey bee, *Apis mellifera*, face numerous challenges that can impact their survival and productivity^1,2^. These challenges are driven by biotic and abiotic stressors such as pests and pathogens, poor nutrition, pesticide exposure, and climatic instability^3^. Beekeeping management is a key aspect of honey bee health because it can help mitigate some of the negative effects resulting from these stressors. For example, low diversity of forage availability around the colony can be mitigated with high-quality supplemental feeding^4,5^, and pests such as *Varroa* mites can be controlled with cultural, mechanical, and chemical control practices^6,7^. With optimal beekeeping management practices, beekeepers can largely maintain healthy and productive honey bee colonies suitable for sustainable beekeeping operations^8,9^. Despite this, annual colony losses continue to be higher than the historical average (~ 15%) in the United States, and beekeepers around the globe continue to seek advice on best management practices to maintain healthy and productive colonies^10–13^.

One of the major challenges that beekeepers face when incorporating different beekeeping practices is that management decisions are constrained by the size of the beekeeping operation and the philosophy of the beekeeper toward chemical treatments^14,15^. For instance, time-consuming practices such as using monthly mite population estimates to determine treatment needs are most often used by small-scale hobbyist operations but are not feasible for large-scale migratory beekeepers (operations above 500 colonies)^15,16^. These constraints result in similar management practices among large-scale beekeepers while small-scale beekeepers vary significantly in the practices that they implement^17^. In particular, beekeepers managing small operations significantly vary in their willingness to intervene in the colony and apply chemicals for pest control^15^. These fundamental differences in the scale of operations and willingness to apply chemicals to colonies (hereafter beekeeping philosophy) are important determinants of the management practices that beekeepers choose to use in their operations and, subsequently, the health and survival of their colonies^17^.

Despite variations in management practices, beekeepers can be grouped under three broad categories of management systems based on their beekeeping philosophies^15,18^ (Table 1). *Conventional management* is based on frequent intervention and application of any available chemical and nutritional supplement to keep colonies alive. This management system is often used by large-scale commercial beekeepers and incorporates the use of synthetic chemicals and antibiotics for pest and disease control. In contrast, *organic management* is based on intervention only as needed and excludes the application of synthetic chemicals or antibiotics to colonies. This management system is common among small and medium-scale beekeepers (a.k.a. sideliners), and it is based on an integrated pest management approach that combines cultural practices with organic-approved chemical treatments (e.g., formic acid, oxalic acid, thymol) for pest control^19,20^. Last, *chemical-free management* is a popular management system, typically among hobbyists, and it is characterized by the absence of chemical applications and the minimal frequency of interventions to the colony^15^. This system strictly relies on cultural practices for pest control (e.g., small cell comb) and the bees’ own defenses against pathogens^21^.

**Table 1:** Summary of differences among the three management systems based on beekeeping philosophy.

	Conventional	Organic^a^	Chemical-free
Use of synthetic chemicals	Y	N	N
Use of soft chemicals	Y	Y	N
Use of cultural controls	Y	Y	Y
Frequency of intervention	High	As needed	Low
Time commitment per colony	Low	Medium	Low

This information is based on results from Underwood et al.^15^. Protocols for each management system developed for this experiment are congruent with these differences and were based on input from advisor beekeeper participants.

^a^The term “organic” refers to the type of management that uses principles of integrated pest management (IPM) and limits in-hive products used for pest control to organic-approved^19,20^. However, the products of these colonies cannot be certified as organic by the USDA due to land-use restrictions^22^.

One of the motivations that chemical-free beekeepers have for avoiding the use of chemicals to control pests and pathogens is that these compounds can trigger trade-offs between the benefits of reducing pests and the risks of negative side effects^23–25^. For example, while some synthetic chemicals are highly effective for *Varroa* mite control, they may have negative fitness consequences for honey bees as they can decrease sperm viability, and alter metabolic responses, cardiac function, and virus tolerance^26–29^. However, one limitation of studies experimentally testing the effects of miticides on honey bee health is that they largely focused on a single treatment. This contrasts with the reality of beekeeping management where the multiple outcomes of risks and benefits of miticide applications occur in the context of numerous other management decisions involved in beekeeping^15^. Therefore, experiments that use a systems approach are necessary to better understand the trade-offs between the risks and benefits of chemical treatments. Despite this, most studies in the literature have investigated the effect of one or two aspects of management at a time.

Another important limitation of the existing literature is the scarcity of studies aiming to identify the long-term effects of different management practices on honey bee health. Most studies investigate the impact of beekeeping practices on colony survival after the first year or follow apiaries over multiple years even when colonies are replaced due to mortality rather than following individual colonies continuously^9,30,31^. First-year colonies developing on bare foundation are different from older multi-year colonies, which translates into different health challenges and economic profits. For example, the drawing of beeswax comb is very energetically expensive, thus limiting the amount of harvestable honey that can be produced^32^. In addition, from an epidemiological perspective, older colonies are more prone to accumulate pests and diseases and likely require different management practices compared to first-year colonies^33^. Thus, there is a need for longitudinal studies that follow individual colonies over several years to determine whether colony health and productivity respond differently to management over time.

Our ability to characterize and quantify colony health is critical for predicting honey bee survival and colony productivity. Thus, the use of standard biomarkers to assess honey bee colony health in longitudinal studies of beekeeping management practices can facilitate the validation of health biomarkers that can indicate declining strength, nutritional status, or stress^34^. Some of the most common biomarkers of honey bee health include levels of *Varroa* mite infestation^35^, viral titers^36^, and the expression of immune genes^37^ that can be strong predictors of colony overwintering success. While *Varroa* mites can be easily seen and monitored, virus levels require sophisticated laboratory methods and equipment^38^. Thus, conducting field studies incorporating both field and laboratory data are essential for providing beekeeper-relevant experimental results.

The goal of this study was to experimentally test the outcomes of three management systems (conventional, organic, and chemical-free; Table 1) on the health and productivity of honey bee colonies over 3 years. Our approach focused on the assessment of several biomarkers of health including levels of parasitic mites (*Varroa destructor*), pathogens (*Vairimorpha ceranae* [formerly known as *Nosema ceranae*]*, V. apis* [formerly known as *N. apis*], Deformed Wing Virus (DWV) and Israeli Acute Paralysis Virus (IAPV)) and the expression of several genes related to immune function and metabolism (*defensin-1*, *hymenoptaecin*, *naked cuticle gene*, and *vitellogenin*). We used pathogen titers and the levels of immune gene expression as health biomarkers to predict survival. We hypothesized that the organic management system would provide the highest benefits to colonies through trade-offs between controlling pests and pathogens while avoiding exposure to synthetic chemicals. Our results indicate that colonies in the conventional and organic management systems show similar survival and honey production, thus providing evidence that managed honey bee colonies require active management of pests but not the regular use of synthetic chemicals to be sustainable.

## Materials and methods

### Development of protocols for management systems

The protocols used in this study to experimentally test differences between three beekeeping management systems were developed through participatory science from a stakeholder group. We met with 30 beekeepers who represented the conventional, organic, and chemical-free management systems to elaborate on the detailed protocols used in this experiment (Table 1, Appendix 1). The protocols included differences in the source of the packages of bees, type of equipment, type and frequency of pest and pathogen treatments, and type and frequency of feeding.

### Experimental design

**Figure 1:**
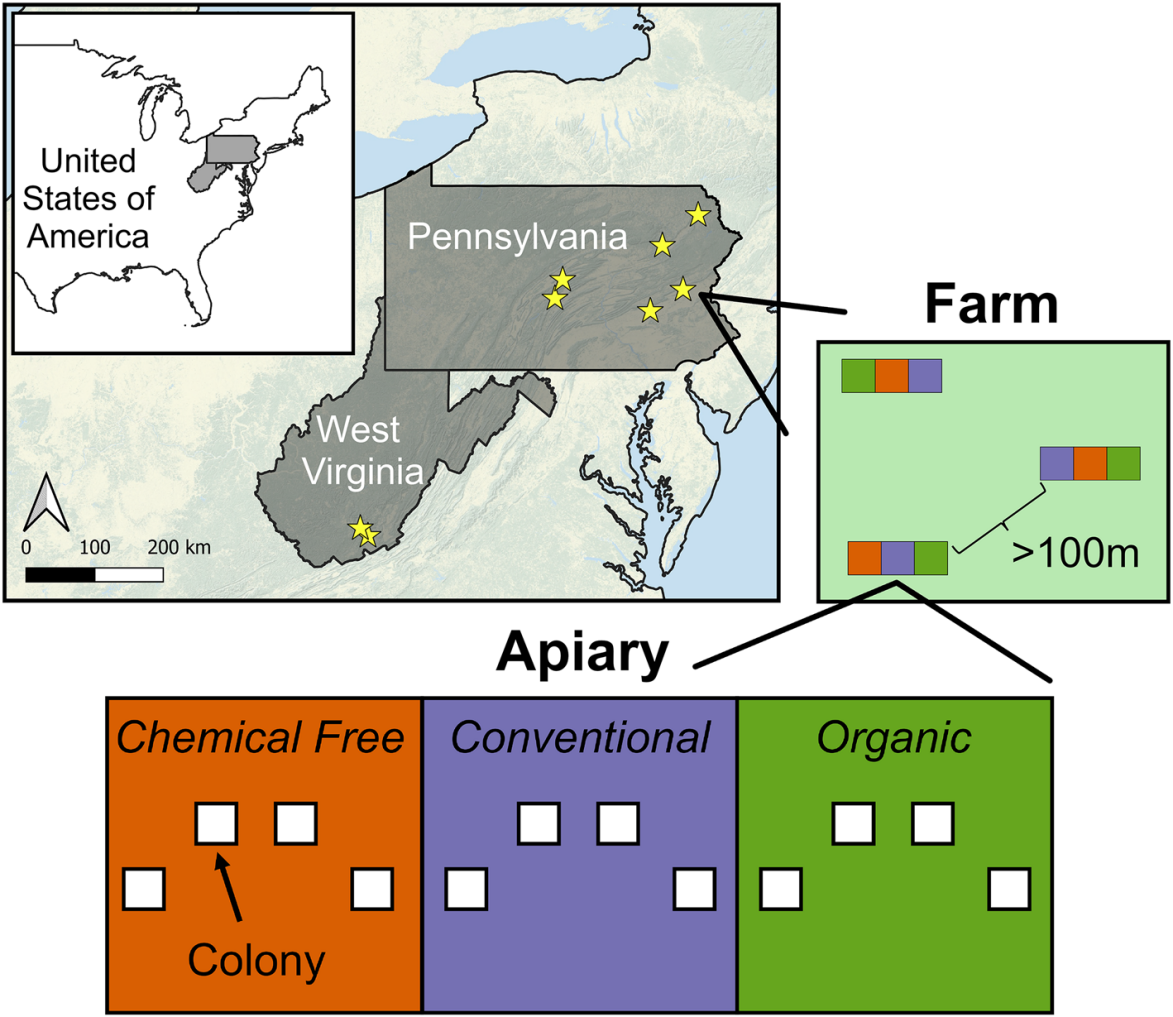
Geographic locations and spatial distribution of farms, apiaries, and colonies. The yellow stars represent the different farm locations. Each farm housed three apiaries at least 100 m apart that contained a total of 12 colonies, 4 in each management system. The spatial arrangement of the different treatments was randomly assigned across apiaries.

We established 288 honey bee colonies at eight certified organic farms: six in Pennsylvania (PA) and two in West Virginia (WV), USA (Fig. 1). Colonies were monitored from April 2018 to April 2021 in PA and from April 2018 to April 2020 in WV. Each of the 8 farms had three apiaries (blocks) that were at least 100 m apart (min = 130, max = 8500, median = 1080, mean = 2080, SD = 2400) and contained colonies of all three management systems (4 colonies per treatment) for a total of 36 colonies per farm. Each apiary was surrounded by an electric fence for protection from bears. Colonies of each treatment were grouped together within each apiary, but the arrangement was randomized across apiaries (Fig. 1).

### Colony establishment and requeening

In late April 2018, we established colonies from 1.5 kg of packaged bees with mated queens purchased from two producers in Georgia, USA. Packages for the conventional and organic management systems were purchased from Roberts Bee Co. and packages for the chemical-free system were purchased from Dixie Bee Supply, because the supplier used small-cell comb and managed colonies organically. Bees from packages were used as comb builders prior to requeening and homogenizing the genetic background of the colonies (see details below). Package bees were established in standard medium Langstroth 10-frame equipment on undrawn plastic foundation for all management systems. For the conventional and organic management systems, the foundation was purchased with standard 5.2 mm hexagonal impressions and a light wax coating. For the chemical-free management system, plastic frames were purchased with small cell 4.9 mm hexagonal impressions and without a wax coating. The all-in-one plastic frames were cut and affixed within standard wooden frames, leaving 2.5 cm of open space on each side of the foundation. The plastic foundation was subsequently coated with beeswax obtained from colonies kept in the desert of Arizona, USA. This wax was pesticide-free because it was collected in an area with no proximity to agricultural chemicals by a beekeeper who does not use in-hive chemicals. In July 2018, we requeened all colonies in the study with sister queens reared and openly mated near Utica, NY (USA). Queens were produced via grafting from a colony that had not been treated for *Varroa* mites for at least 7 years. After the colonies were established and requeened, no other bees or queens were brought into the study. In order to keep the colony density at 12 per apiary, we made splits from surviving colonies and placed them in empty spaces. These colonies were maintained using the management system of their origin. For apiaries where mortality in the chemical-free system was very high and splitting could not make up the difference, we filled out those spaces with colonies in the organic or conventional management systems. These replacement colonies were monitored and managed according to our protocols but were not included in any analyses or results presented. Only colonies that survived continuously were included in our dataset.

### Management details

**Figure 2:**
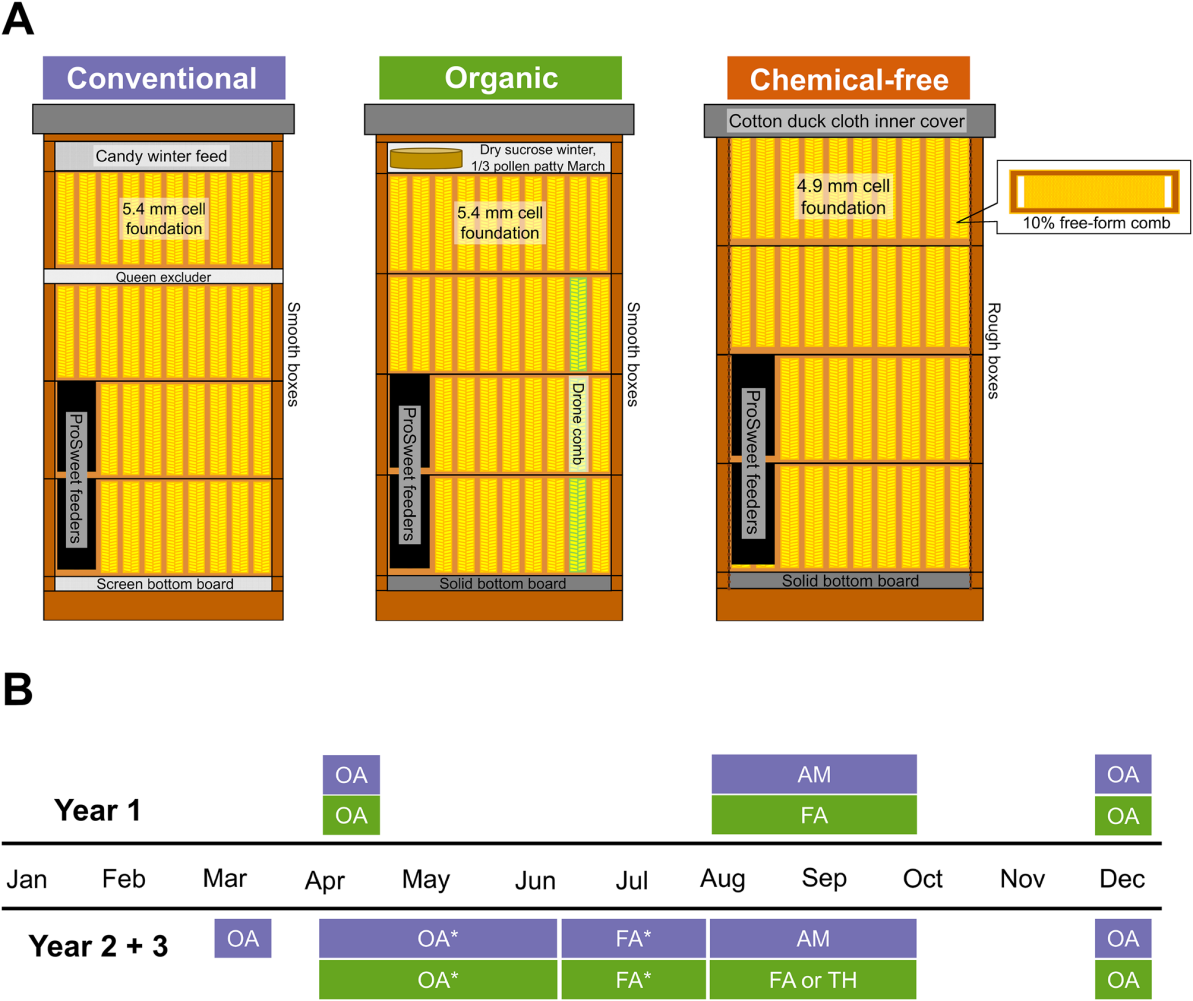
Details of the equipment and treatment applications for each management system. (**A**) The figure depicts a cross-sectional view of each system and shows the equipment utilized in each management system. (**B**) The treatment timeline shows the different types of treatment used including oxalic acid crystal (OA), amitraz (AM), formic acid (FA), or thymol (TH), during year 1 (top) and years 2 and 3 (bottom) for conventional (blue), organic (green), and chemical-free (orange). See Table 1 and Appendix 1 for management details. Asterisks (*) indicate that the treatment was only applied if a threshold of 1% was reached.

The management systems varied in equipment, chemical treatments for parasitic mites, and winter feed (Table 1, Fig. 2, Appendix 1). In brief, colonies in the conventional management system had screened bottom boards, a queen excluder between the third medium box and the honey supers, were treated each fall with amitraz (ApiVar, Veto-Pharma), and were provided candy boards with 3% protein as their emergency winter feed. Colonies in the organic management system had solid bottom boards, did not have queen excluders, were treated with organic-approved chemicals^9,22^ in rotation, employed drone brood removal, and received granulated sucrose as their emergency winter feed. Last, colonies managed under the chemical-free system had solid bottom boards, a cotton duck cloth inner cover, did not have queen excluders, were never treated for mites, and did not receive emergency winter feed (granulated sucrose) unless starvation was imminent. Emergency winter feed was put in place in January of each winter in the organic and conventional systems, but only as needed in the chemical-free system.

### Colony overwintering survival and honey production

We inspected colonies every 2 weeks from March through October in 2018 and 2019 and every 3 weeks in 2020 due to COVID-19 pandemic travel restrictions. Colonies were also visited monthly in the cold months, November through February. Colony survival of all original colonies was recorded throughout the multiple years of the study. Because the highest number of colony losses occurs over the winter, summer losses were not included in the data analyses. Overwintering survival was assessed based on the number of colonies that were alive in October and remained alive in April of the following year. To determine honey production for each apiary, marked supers were individually weighed before and after honey extraction to determine the amount of honey produced by each colony each year of the study. Honey production data were pooled across colonies of the same management system by apiary for data analysis.

### Pests and pathogens

Each year in October, we quantified the abundance of key honey bee pathogens that significantly impact colony health including *Varroa* mites, *V. ceranae, V. apis,* (formerly *Nosema ceranae* and *Nosema apis*, respectively^39^), DWV and IAPV. For *Varroa* mite quantification, we used alcohol washes of approximately 300 bees and counted the number of mites that were removed from the bees during this procedure. All other pathogens were quantified via quantitative reverse-transcription PCR (qRT-PCR) using previously developed primer sequences (Table S1). Abundance of *V. ceranae* and *V. apis* were estimated from DNA extracted using a phenol:chloroform extraction from 30 worker abdomens in pools of five abdomens for a total of six composite samples per colony per sampling period. qRT-PCR was performed as previously described^40^ to quantify the infection levels of each of the pathogens. The RNA viruses DWV and IAPV were quantified from abdomens of a different pool of 30 bees per colony. Tissue was homogenized using Chaos buffer, and RNA was extracted using the Zymo Quick-RNA Microprep Kit following the manufacturer’s protocol. cDNA was synthesized from 2 µg of RNA using random primers and MultiScribe RT, following the manufacturer’s protocol (Applied Biosystems, Foster City, CA). qRT-PCR reactions were carried out in 384 well plates using a QuantStudio 5 Real-Time PCR System (Applied Biosystems). Each well contained 5 µl PowerUp™ SYBR™ Green Master Mix (Applied Biosciences, Thermofisher Scientific, Waltham, MA), 0.25 µl of each of the forward and reverse primers (10 µM), 2.5 µl nuclease-free H_2_O, and 40 ng cDNA template. Reactions were carried out under the following conditions: 60 s at 95 °C for initial denaturation, then 40 cycles of 15 s at 95 °C for denaturation, and 30 s at 60 °C for annealing and extension. Data collection was followed by a melting curve analysis of 15 s at 95 °C, 60 s at 60 °C, and 1 s at 95 °C to determine the specificity of amplification products. All reactions were run in triplicate and each plate included negative controls of nuclease-free water for each set of primers. The Ct value for each sample was determined by taking the mean of the three technical replicates. Any technical replicates that raised the standard deviation of the three replicates above 1 were removed. The cutoff value for measurable expression was a Ct value of 35 or higher. We subtracted the Ct value of the reference gene from the Ct value of target genes to generate ΔCt values for each sample. The sample with the highest ΔCt value for each virus was used as a baseline to calculate the ΔΔCt value^41^. We used the ΔΔCt method for virus quantification to facilitate data transformation and meet assumptions of normality for downstream data analysis.

### Gene expression

We quantified the expression of four genes as biomarkers of honey bee health^34,36,37,42^. The gene *vitellogenin* (*vg*) was selected because higher levels of expression of this gene have been identified as a biomarker as it is associated with *A. mellifera* colony overwintering success^37^. The antimicrobial peptide genes *hymenoptaecin* (*hym*) and *defensin-1* (*def1*) were selected as indicators of the immune response to infection^36^, and the *naked cuticle gene* (*nkd*) was used to quantify immune regulation function of the *Wnt* pathway^42^. *Nkd* down-regulates the *Wnt* pathway and it has been hypothesized as a biomarker of reduced immune response associated with higher viral titers in *A. mellifera*^42^. Gene expression was quantified from the same RNA extractions and qRT-PCR protocol used for the viral pathogens.

### Statistical analysis

To investigate the effect of management system on colony health, we averaged data across years and colonies within each apiary to get one value per treatment, per apiary, resulting in 72 data points (24 apiaries × 3 treatments). For honey production, we calculated the sum of total production for each apiary as this value reflects the total production of honey per apiary. We log or log + 1 transformed all the qPCR variables (*Vairimorpha* spp., DWV, IAPV, *hym*, *def1*, *vg*, *nkd*) because they were strongly left-skewed and contained a small number of extremely large values. *Vairimorpha apis* and IAPV were excluded from analyses because of the large number of zeros in these variables. We did not transform *Varroa* mite numbers to facilitate the interpretation of results. In addition, the output of the analyses was similar between raw and log-transformed data. We used mixed effects linear models using the ‘lmer’ function in the lme4 package to quantify the effects of management system on all response variables using ‘farm’ (a factor with 8 levels) as a random effect [Model syntax: lmer(y ~ management system + (1|farm)]^43^. To investigate the effect of management system, year, and their interaction, we included a continuous variable for ‘year’ to account for repeated measures and ‘farm’ as random effect [Model syntax: lmer(y ~ management system * year + (year|farm)]. We calculated p-values from the models using the ‘Anova’ function in the car package^44^ with type 3 sums of squares and F-statistics calculated using Kenward-Roger approximation of degrees of freedom. To identify differences between the management systems, we conducted post-hoc Tukey HSD tests using the ‘emmeans’ function in the emmeans package^45^. We estimated the effect sizes of the organic and conventional treatments compared to the chemical-free system control calculated as (treatment—control)/control * 100. Last, we used path analyses to estimate the effect of management systems on biomarkers of health (pests, pathogens, and gene expression) and colony survival using the ‘sem’ function of the lavaan package^46^. To normalize and standardize the data, we transformed all variables except survival and scaled them [mean = 0; standard deviation = 1]. We then created a contrast for management system that compared organic (ORG) and conventional (CON) treatments to chemical-free (CF) treatment because this contrast explained the most variation and ORG and CON treatments on their own had very similar results to each other. To see how relationships varied over the course of the study, we fit a path analysis for 2018 (year 1) and 2019–2020 (years 2 and 3) separately. We combined 2019 and 2020 because of the greatly reduced number of data points in 2020.

## Results

### Colony survival and honey production

**Figure 3:**
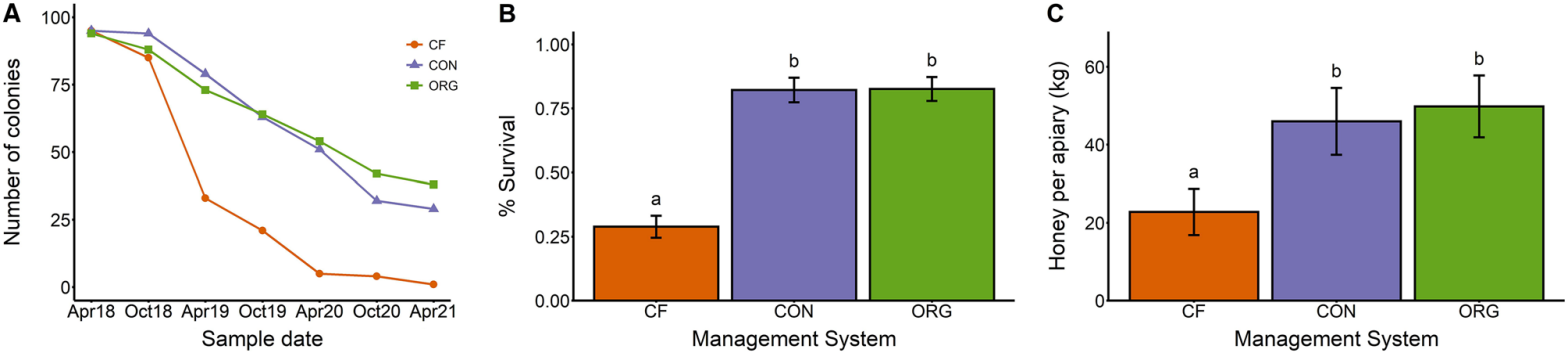
Summary of the effects of management system on the (**A**) number of colonies, (**B**) overwintering survival, and (**C**) honey production per apiary over the three years of the experiment. The three tested management systems were chemical-free (CF, orange), conventional (CON, blue), and organic (ORG, green). Columns with different letters are significantly different from each other (Tukey HSD *p*-value < 0.05). The number of colonies after April 2020 does not include colonies from apiaries in West Virginia.

Colonies with chemical-free management showed reduced survival and honey production compared to those with organic and conventional management. At the beginning of the study there were 96 colonies of each management system and after 3 years only 1 chemical-free colony remained while 29 and 38 conventional and organic colonies survived, respectively (Fig. 3A). Organic and conventional management systems both increased survival by over 180% compared to chemical-free management (Table 2, Fig. 3B). Organic and conventional management also increased total honey production across 3 years, with organic management increasing production by 118% and conventional increasing production by 102% (Table 2, Fig. 3C). The effect of management on honey production varied by year (Table S3) with no significant differences among treatments in the first year, and the chemical-free producing less honey in years 2 and 3 (Fig. S2). Organic and conventional management systems did not differ in survival or honey production (Table S2, Fig. 2).

**Table 2:** Results of mixed linear models showing effects of the management systems on the different response variables (survival, honey production, pests, pathogens, and gene expression) averaged across 3 years.

Variable	F	*p*
Survival	57.57	< 0.0001
Honey production	5.52	0.006
*Varroa* mites	52.76	< 0.0001
*Vairimorpha ceranae*	4.05	0.022
DWV	15.16	< 0.001
*def1*	28.40	< 0.001
*hym*	22.13	< 0.001
*nkd*	8.52	0.001
*vg*	2.17	0.122

Farms were used as random effects. Degrees of freedom for all tests are ndf = 2 and ddf = 62.

### Parasites and pathogens

**Figure 4:**
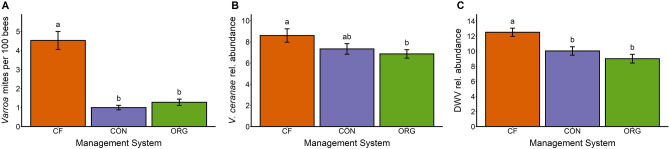
Summary of the differences in the (**A**) number of *Varroa* mites per 100 bees, (**B**) relative abundance of *V. ceranae*, and (**C**) relative abundance of DWV in colonies under the three treatments. The three tested management systems were chemical-free (CF, orange), conventional (CON, blue), and organic (ORG, green). Columns with different letters are significantly different from each other (Tukey HSD *p*-value < 0.05).

Organic and conventional management both reduced *Varroa* mites, *V. ceranae*, and DWV levels. *Varroa* mites were found in 92% of colonies, but organic and conventional management reduced mite abundance by 72% and 78%, respectively, relative to the chemical-free system (Table 2; Fig. 4A). We found a significant interaction between management and year due to increases in *Varroa* mites over time (Table S3, Fig. S2). Particularly, chemical-free colonies showed the highest levels of *Varroa* mites over the 3 years of the study with an average of 4.5 mites per 100 bees (Fig. S4). *Vairimorpha ceranae* was detected in 98% of colonies. We found moderate evidence that organic management reduced *V. ceranae* levels by 20% (Tukey HSD, *p*-value = 0.02; Fig. 4B) and weak evidence that conventional management reduced levels by 15% (Tukey HSD, *p*-value = 0.12; Fig. 4B). However, these effects were not significant when year was included as a fixed effect in the model (Table S3, Fig. S2). Deformed Wing Virus (DWV) was affected by management system (Table 2) with organic and conventional treatments reducing DWV relative abundance by 28% and 20% (Fig. 4C). *Vairimorpha apis* was detected in only 26% of colonies and management system did not have an effect on its relative abundance (F_2,62_ = 0.27, *p*-value = 0.76), so was not included in other analyses.

### Gene expression

**Figure 5:**
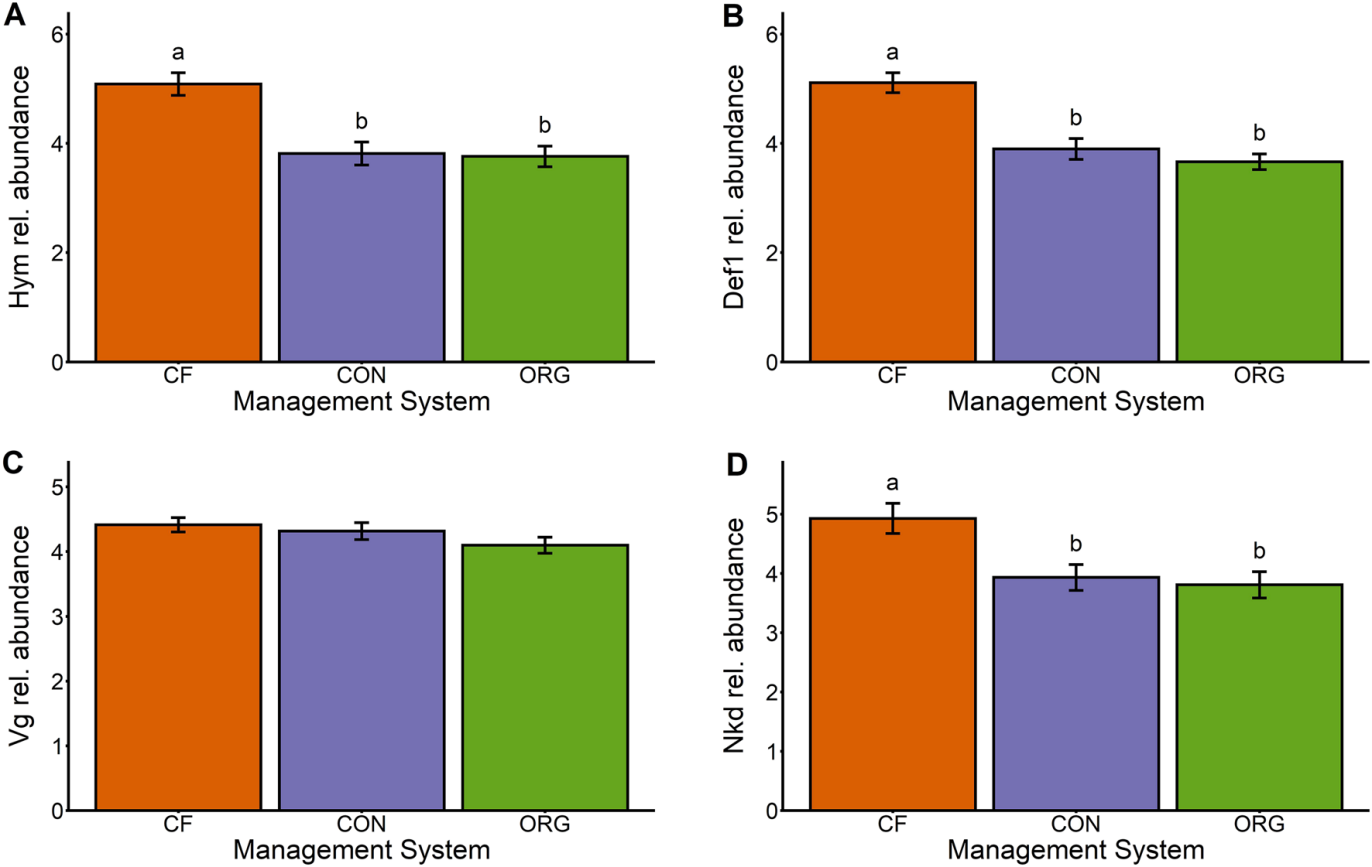
Summary of the differences in the expression of (**A**) *Hymenoptaecin* (*hym*), (**B**) *Defensin*-1 (*def1*), (**C**) *Vitellogenin* (*vg*), and (**D**) *Naked cuticle gene* (*nkd*) in colonies under the three tested management systems; chemical-free (CF, orange), conventional (CON, blue), and organic (ORG, green). Columns with different letters are significantly different from each other (Tukey HSD *p* < 0.05).

The expression of *hym*, *def1*, and *nkd*, but not *vg*, varied across the different management systems. The organic and conventional management systems reduced the expression of *hym*, *def1*, and *nkd* between 20 and 28% relative to chemical-free (Table 2, Fig. 4A,B,D). In all cases, there were no significant differences between organic and conventional treatments (Table S2, Fig. 5). Expression of *vg*, however, was not impacted by management system (Table 2, Fig. 5C). Levels of expression varied across years (Fig. S3), but we did not detect a significant interaction between management system and year (Table S3).

### Predictors of overwintering survival

**Figure 6:**
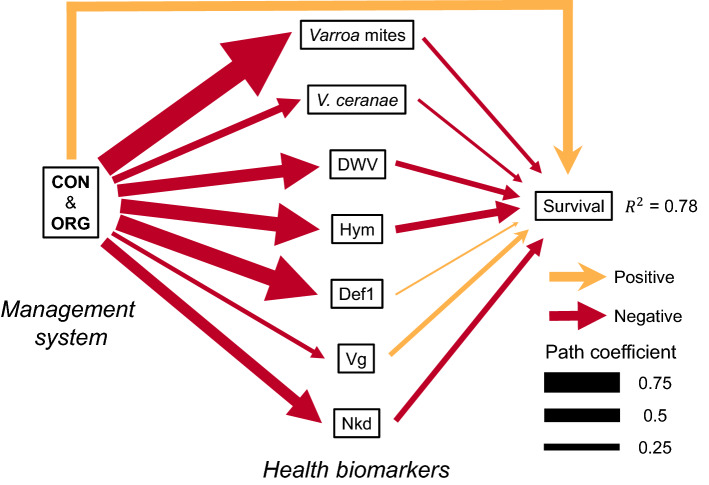
Path analysis showing the effects of management system (left) on health biomarkers (middle) and how they impact honey bee colony winter survival (right). Negative paths coming from the “CON & ORG” management system box indicate that the conventional and organic management systems reduced levels of variables compared to chemical-free treatments. Data used in the model treated apiaries as replicates (N = 72) and was averaged across 3 years of the study. Colors indicate positive (orange) and negative (red) effects. The width of the arrows is scaled by the path coefficients (ranging between 0 and 1) and indicates the strength of the association varying from low (thin) to high (thick). The arrow connecting management and survival shows the direct effect that is not explained by the intermediate health biomarkers.

The variables included in the path analysis explained 78% of the variation in colony overwintering survival (Fig. 6). The strongest single predictor of survival was the direct effect of management system (r = 0.36), with organic and conventional treatments associated with increased colony survival compared to chemical-free. Honey bee pests and pathogens (*Varroa* mites, *V. ceranae,* and DWV) were all negatively associated with survival with DWV having the largest effect (r = − 0.2), *V. ceranae* the smallest (r = − 0.1) and *Varroa* mites had an intermediate value (r = − 0.15). Increases in the expression of the genes *hym* (r = − 0.29) and *nkd *(r = − 0.21) were also associated with reductions in survival. In contrast, *vg* was positively associated with survival (r = 0.18). *Def1* had a weak non-significant association with survival. The effects of management on survival mediated by health biomarkers corroborates that organic and conventional management systems are associated with lower pests and pathogens, and reduced immune gene expression. The impact of management system on *Varroa* mites was the largest (r = − 0.81), while the effects on *vg* and *V. ceranae* were the weakest, and the other variables had intermediate values. Despite the consistency in the major direct effects of management on colony survival, the path analysis for the first year of the study (2018) showed stronger effects of colony variables on survival during the winter of 2018–2019 than on survival during the winters of 2019–2020 and 2020–2021 (Fig. S5).

## Discussion

Our experimental study investigates the long-term effects of three beekeeping management systems (conventional, organic, and chemical-free) on honey bee survival and productivity. With a model including management system and seven biomarkers of health, we were able to explain 78% of the variation in colony survival. These results indicate that despite the myriad of biotic and abiotic stressors impacting honey bees^3^, beekeeping management is the most important factor associated with colony health and productivity for stationary honey-producing colonies. Overwintering losses for the conventional and organic management systems averaged 23% over the 3 years of the study. This value is lower than the overwintering losses reported by beekeepers in the United States, who on average report around 40% losses annually^47^. Results from this study highlight the importance of incorporating data-driven management recommendations into beekeeping operations, which remains one of the major challenges for a more sustainable industry. Beekeepers that use a chemical-free management system oppose the use of chemical miticide treatments and interventions in the colonies because of the potential negative impacts of these treatments on the bees. Our results demonstrate that the benefits of miticide treatments outweigh their detrimental effects on honey-producing operations. Indeed, the lack of miticide treatments led to unhealthy colonies that were five times more likely to die than colonies treated for mites. While there is substantial evidence supporting that honey bee populations can persist in unmanaged conditions without *Varroa* mite treatments^48–53^, our results demonstrate that the chemical-free system is not suitable for honey-producing beekeeping operations. Colonies under the chemical-free management system experienced mortality of 70% yearly despite the use of queens from a local colony that had not been treated with miticides for 7 years. These results indicate that monitoring mite populations is critical for the survival of honey-producing operations, and chemical treatments are imperative when mite levels rise above a 1–2% threshold to avoid high colony overwintering mortality^54^.

One important finding of this study is that we demonstrate that the organic and conventional management systems performed comparably in terms of survival and honey production, suggesting that the organic system can be considered a sustainable management approach for small to mid-size stationary honey-producing operations. This is the first study to experimentally test the suitability of organic approaches for honey bee colony management. Our organic management system relies on threshold-based applications of organic miticides, among other practices^15^, which are approved for use in organic apiculture^9,22^. For *Varroa* mites, colonies in the conventional system showed the lowest number of mites of all systems but, on average, mites were under threshold levels (below 2 mites per 100 bees) in both the conventional and organic systems (X_CON_ = 1 mite per 100 bees; X_ORG_ = 1.28 mites per 100 bees; X_CF_ = 4.52 mites per 100 bees; Table S2). In contrast, the lowest levels of *V. ceranae* were found in colonies in the organic management system (Table S2). Taken altogether, these results suggest that not only is the organic system suitable for a sustainable beekeeping industry but that the use of threshold-based criteria for the application of organic miticides can have positive effects on colony health. It is important to note, however, that the products of these colonies cannot be marketed as organic unless there is sufficient organic habitat to support the exclusive collection of pesticide-free pollen and nectar^22^. Currently, the recommendations for organic beekeeping in the United States include a pesticide-free forage zone of a 3 km radius with an additional surveillance radius of 3.4 km around the bee yard. Future research should investigate the feasibility of supporting colonies in pesticide-free foraging habitat placed in smaller, high-quality certified organic areas.

The use of several biomarkers of health in this study indicates that *hym,* DWV, *nkd*, and *vg* are adequate indicators of colony health^34^. However, when accounting for all variables, management system was the strongest single predictor of survival (r = 0.36; Fig. 6) even though many of the variables on their own are strongly correlated with survival (Fig. S4). This suggests that it is the aggregate effect of management system on many factors that is an important determinant of colony survival. After accounting for the effect of management system, *Varroa* mite levels were a weak predictor of colony survival^55^ (r = 0.15; Fig. 5). However, without intervention, *Varroa* mite loads in the colonies in the chemical-free system increased dramatically (Fig. S4) and were strongly associated with lower survival (Fig. S6). Surviving colonies in the chemical-free treatment showed high mite levels and reared offspring in the presence of high mite loads, which likely had a great impact on their overall health and survival through the winter^36^. We found associations between *Varroa* mite levels and the other biomarkers of health that we assessed (Fig. S6). *Varroa* mite levels were strongly positively correlated with DWV viral titers and the expression of the antimicrobial peptides *def-1* and *hym* (r > 0.65; Fig. S6). The role of *Varroa* mites in colony overwintering losses, and the spread and activation of viral pathogens has been well established^1,56–58^. Therefore, it is not surprising that management systems that control mites also show dramatic decreases in viral titers^59,60^. In contrast, *vg* levels were not affected by management, a result that is consistent with its role as a biomarker of the nutritional status of the colony^37^. The expression levels of *nkd*, which down-regulates the *Wnt* pathway and reduces overall immune response in *A. mellifera,* were positively associated with *Varroa* levels corroborating previous studies suggesting that this gene may be a reliable biomarker of health^42^. Overall, we conclude that, of the biomarkers quantified in the laboratory, DWV titers, *hym,* and *nkd* were the most strongly and negatively associated with colony survival. In contrast, *vg* expression was positively associated with survival.

In the present study, we used a holistic systems approach to investigate the collective impacts of management practices on honey bee health and productivity. This experimental approach is particularly important for the application of our results into beekeeping operations, as decisions in beekeeping are rarely taken in isolation. Thus, our study differs from the multitude of experiments that have investigated how different aspects of beekeeping management (e.g., chemical *Varroa* mite control measures, equipment choices, and type of feeding) individually impact honey bee survival and productivity^6,19,61–63^. Previous studies similar to ours focusing on assessing the impact of beekeeping management on honey bee health have primarily used survey data to address similar questions, and have found comparable results regarding the key role that beekeeping management plays in honey bee survival^8,14,64^. However, this is the first study to experimentally assess how the integration of different practices into management systems impacts colony health and sustainability over multiple years in a controlled experimental setting. The well-replicated nature of this study, which included 8 farms distributed across two states in the United States, also provides strong support for our results despite differences in environmental variables, such as landscape quality. However, in order to validate the generality of our protocols, future experiments outside of the Mid-Atlantic region of the United States will be necessary. Our prediction is that some aspects of our protocols will need to be adjusted due to regional differences in climate, floral phenology, and the presence of additional pests. Our results provide strong evidence supporting that organic beekeeping practices are as effective as the practices used in the conventional system while avoiding the use of synthetic pesticides to control pests and pathogens inside the hive. Beekeeping management is a multifaceted problem that requires complex approaches to problem-solving, but it is critical for effectively maintaining healthy honey bee colonies.

## Supplementary Information

Supplementary Information.

## Data Availability

All data generated and analyzed for the present study will be included in the published article upon acceptance.
