# Supplementary material for: A longitudinal experiment demonstrates that honey bee colonies managed organically are as healthy and productive as those managed conventionally
Source: Sci Rep. 2023 Apr 13;13:6072. doi: 10.1038/s41598-023-32824-w (PMC10100614; doi:10.1038/s41598-023-32824-w)
Supplement: Supplementary file 1 — Supplementary Information. [file 41598_2023_32824_MOESM1_ESM.docx]

**Supplementary Figures and Tables**


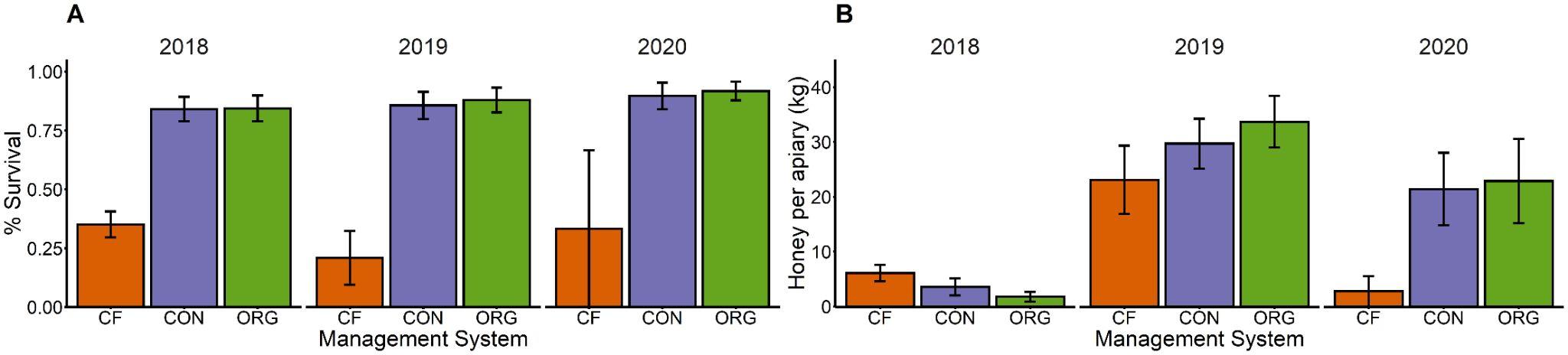


**Figure S1.** Summary of the effects of management system on (A) overwintering survival, and (B) honey production for each of the three years of the experiment. The three tested management systems were chemical-free (CF, orange), conventional (CON, blue), and organic (ORG, green).


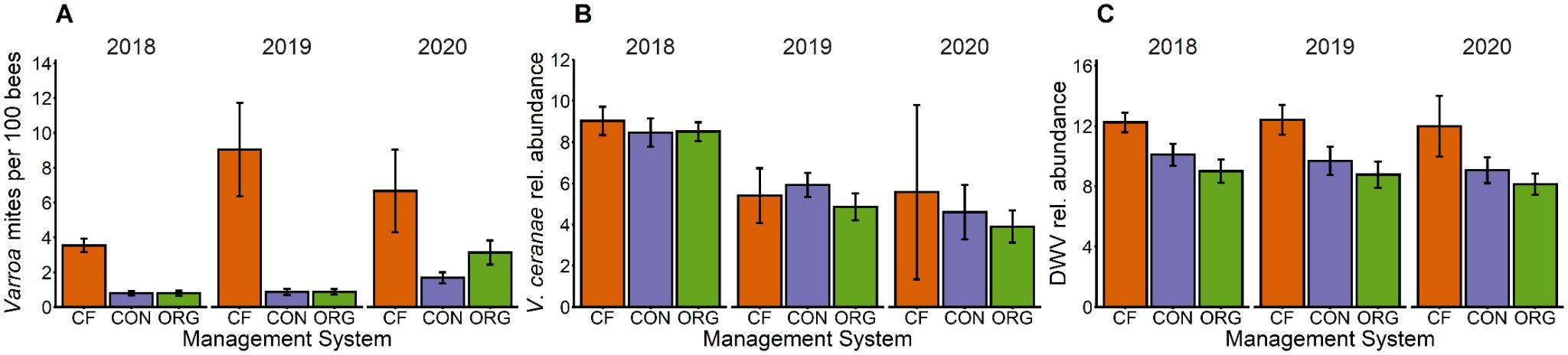


**Figure S2.** Summary of the differences in (A) number of *Varroa* mites per 100 bees, (B) relative abundance of *V. ceranae*, and (C) relative abundance of DWV in colonies under the three different management systems (CF: chemical-free, CON: conventional, ORG: organic) during the three years of the experiment.


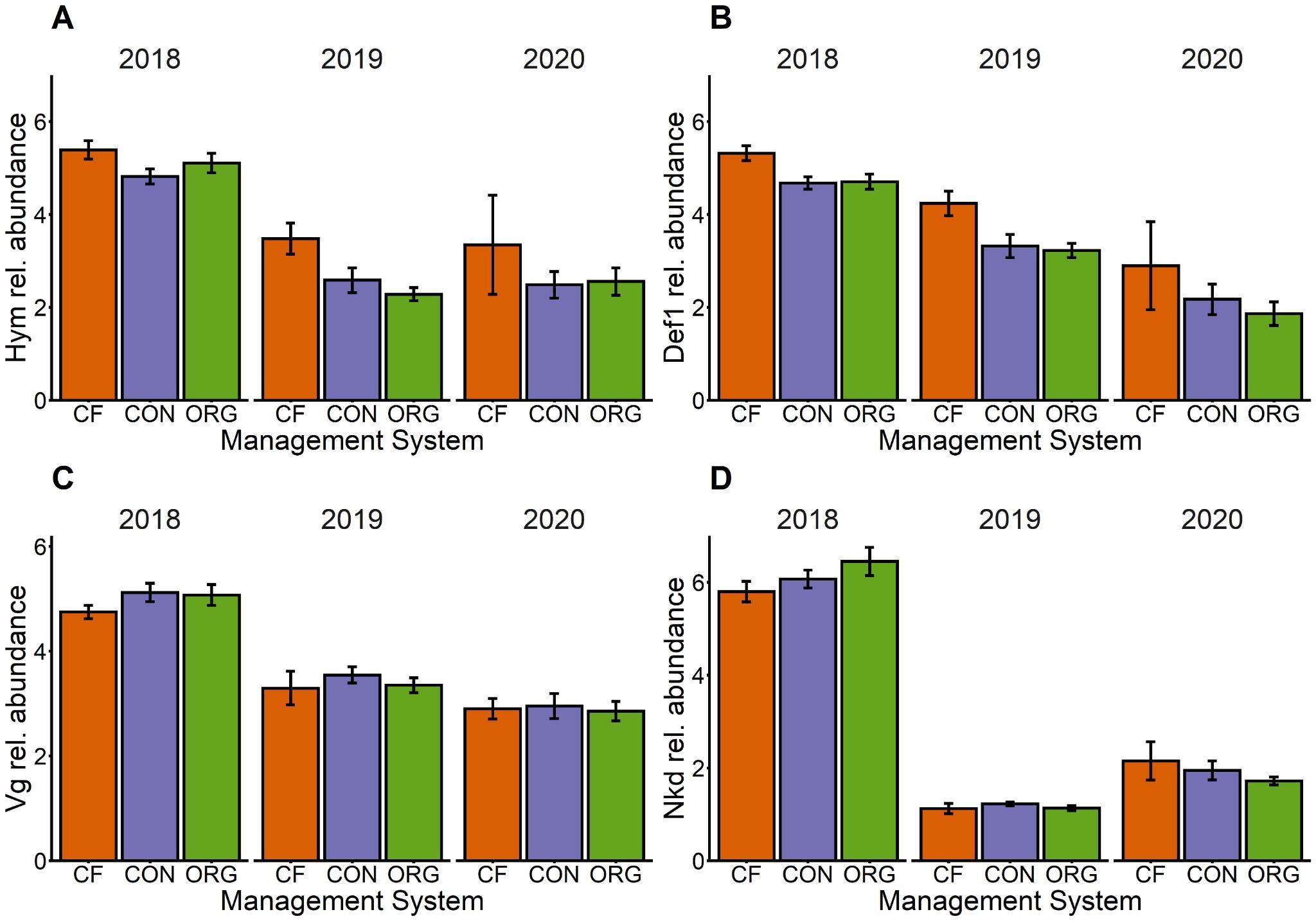


**Figure S3**. Summary of the differences in expression of (A) Hymenoptaecin (*hym*), (B) Defensin-1 (*def1*), (C) Naked cuticle gene (*nkd*), and (D) Vitellogenin (*vg*) per year in colonies under the three different management systems (CF: chemical-free, CON: conventional, ORG: organic).


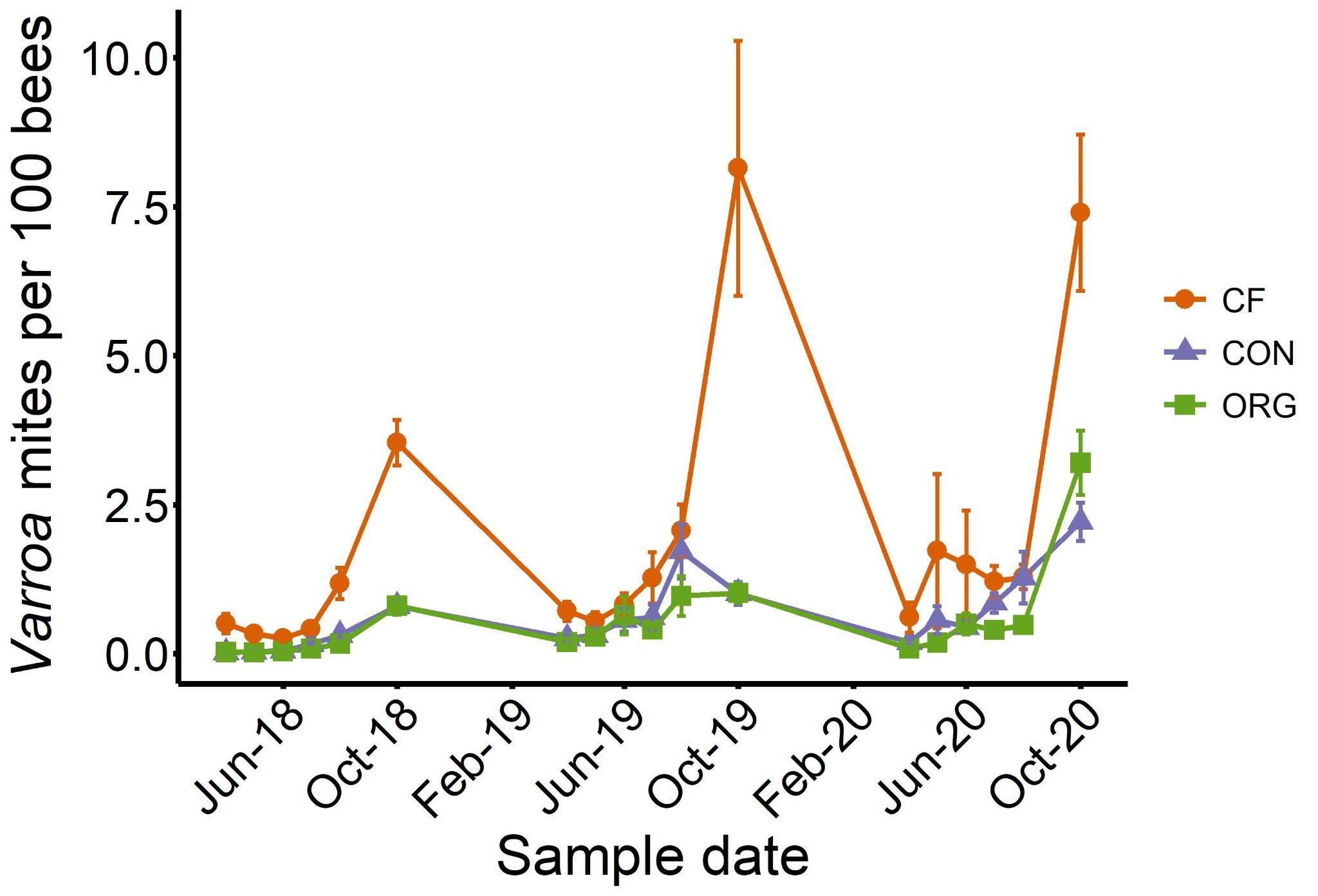


**Figure S4.**  Changes in *Varroa* number per 100 bees for colonies in three tested management systems were chemical-free (CF, orange), conventional (CON, blue), and organic (ORG, green). Each point shows the average *Varroa* numbers for all surviving colonies during the three years of the experiment. Bars indicate standard errors.


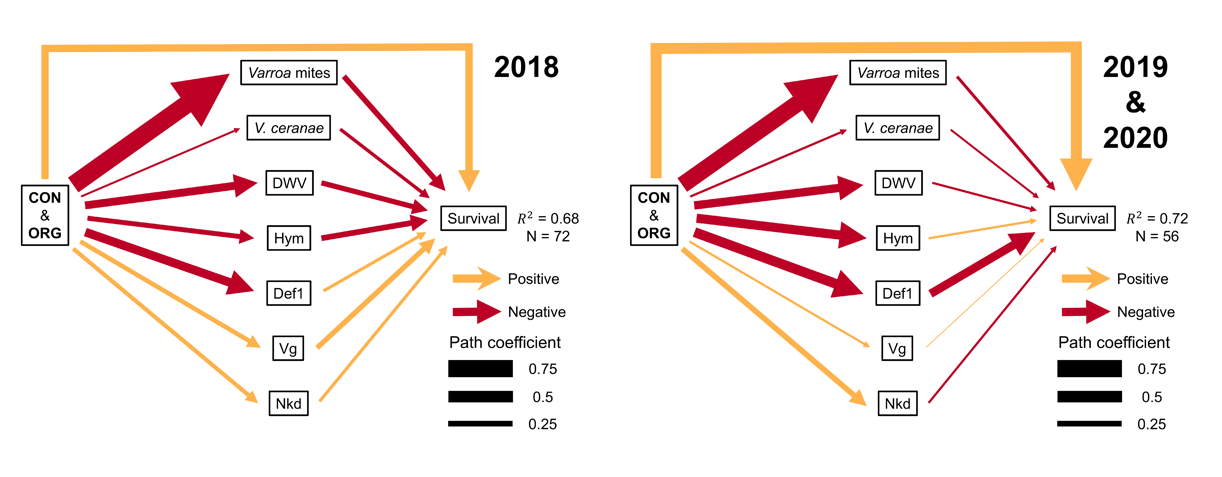


**Figure S5.** Path analysis showing the effects of management system on health biomarkers (middle) and how they impact honey bee colony winter survival (right) for colonies in 2018 and ‘2019 and 2020’. Colors indicate positive (orange) and negative (red) effects. Width of the arrows indicate the strength of the association varying from low (thin) to high (thick) associations. In 2018, all colony variables except *def1* had significant effects on survival whereas in 2019-2020 *def1* was the only significant path. In 2018, *def1* had weak positive association with survival (r = 0.14) while in 2019-2020 it had a strong negative effect (r = -0.37).


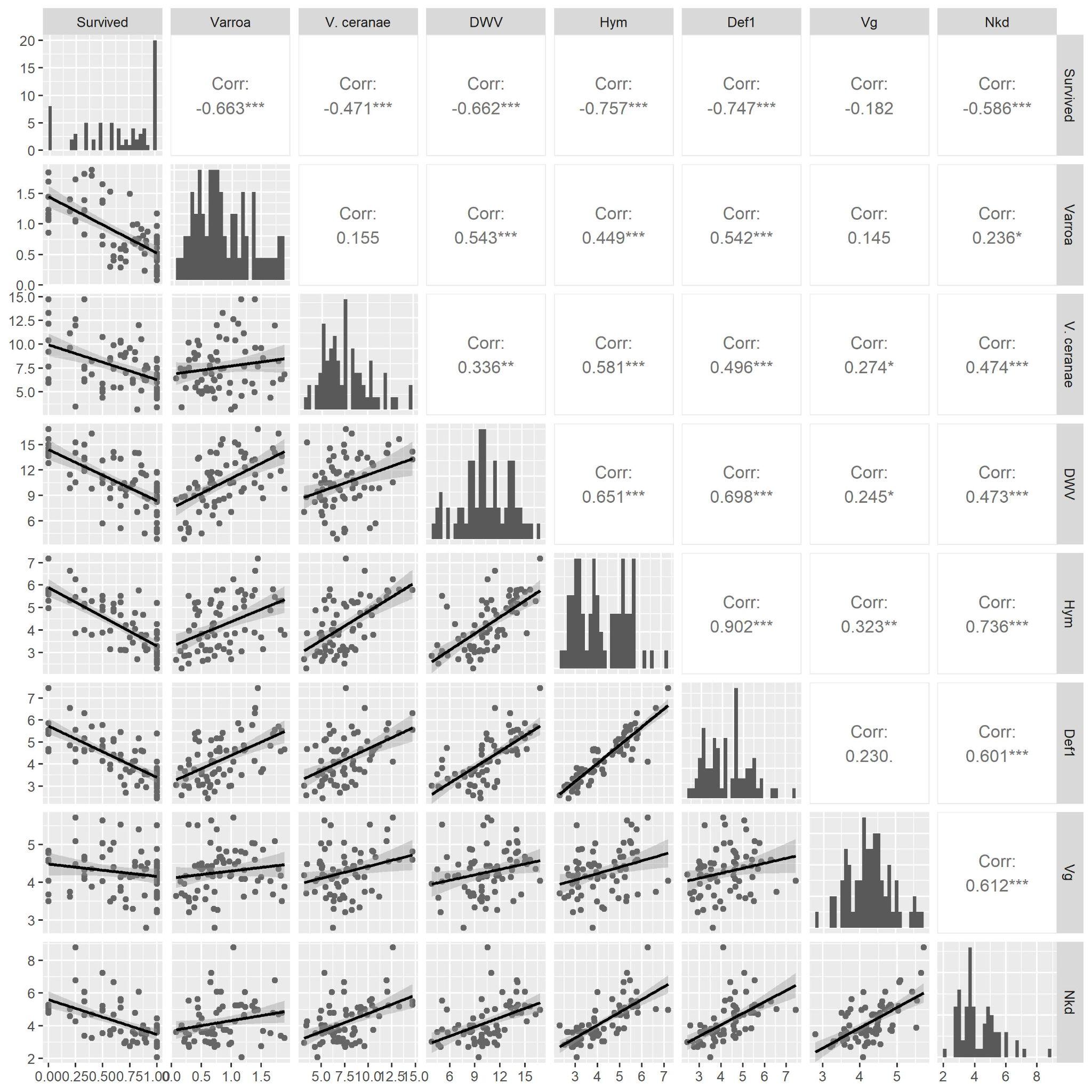


**Figure S6.** Pairwise correlations between all continuous variables in the study. Scatter plots on the bottom half show the relationship between each pair of variables along with the best fit linear line. The diagonal shows a histogram of each variable, and the top half shows Pearson correlation coefficients.

**Table S1.** Information about sequences of forward and reverse primers used for the quantification of pathogens and immune genes through quantitative PCR.

| **Target** | **Gene Category** | **Primer set (5’- 3’)** | **Reference** | **GenBank Reference Sequence no.** |
| --- | --- | --- | --- | --- |
| Deformed Wing Virus | Virus | Forward: GTTTGTATGAGGTTATACTTCAAGGAG  Reverse: GCCATGCAATCCTTCAGTACCAGC | Ryabov et al. 2014 | AJ489744 |
| Israeli Acute Paralysis Virus | Virus | Forward: GTTGGATGATAGGTCCACCCC  Reverse: TCAAGTGTCGGTTTTCGGTC | Jones et al. 2021 | NC_009025.1 (Maori et al., 2007) |
| Vitellogenin | Nutritional status biomarker | Forward: GCA GAA TAC ATG GAC GGT GT  Reverse: GAA CAG TCT TCG GAA GCT TG | Lourenco et al. 2012 |  |
| Hymenoptaecin | Immune Gene (AMP) | Forward: ACAATGGATTATATCCCGACTCGT  Reverse: CAATGTCCAAGGATGGACGAC | Vannette et al. 2015 | FJ546166 |
| Defensin-1 | Immune Gene (AMP) | Forward: GGCTGCACCTGTTGAGGAT  Reverse: TGTCCTTTGAATGAGAGAAGGTCA | Vannette et al. 2015 |  |
| Naked cuticle gene | Immune Gene (Wnt) | Forward: AGGATGACGGTGAAAATGCG  Reverse: ATTAGTCGTGAGGAGAGGCG | Li et al. 2016 | NM_001011616 |
| Ef1-alpha | Endogenous reference | Forward: GGAGATGCTGCCATCGTTAT  Reverse: CAGCAGCGTCCTTGAAAGTT | Lourenco et al. 2008 |  |

**Table S2.** Summary statistics (mean and standard deviation) for all variables in the study across the three management systems. Variables were averaged across the three years using apiary as replicates (N=72). Posthoc results are from the same models that are summarized in Table 2 and are Tukey HSD tests of mixed effects models testing the effects of management system on each response variable. The three columns show the posthoc test of the contrasts between each management system, for example, “CF-CON” is testing differences in means between chemical-free and conventional systems. P values <0.05 are shown in bold.

|  | **Summary Statistics** | | | | | | **Posthoc Tests** | | |
| --- | --- | --- | --- | --- | --- | --- | --- | --- | --- |
|  | **CF** | | **CON** | | **ORG** | | **CF-CON** | **CF-ORG** | **CON-ORG** |
| **Variable** | *Mean* | *SD* | *Mean* | *SD* | *Mean* | *SD* | *P* | *P* | *P* |
| Survival | 0.29 | 0.21 | 0.82 | 0.23 | 0.83 | 0.23 | **<0.001** | **<0.001** | 0.997 |
| Honey production | 22.77 | 29.08 | 45.98 | 42.04 | 49.81 | 38.83 | **0.028** | **0.009** | 0.901 |
| *Varroa* mites | 4.52 | 2.31 | 1 | 0.57 | 1.28 | 0.83 | **<0.001** | **<0.001** | 0.748 |
| *V. ceranae* | 8.6 | 3.11 | 7.33 | 2.45 | 6.86 | 1.94 | 0.119 | **0.021** | 0.739 |
| DWV | 12.51 | 2.65 | 10.03 | 2.74 | 9.01 | 2.84 | **0.001** | **<0.001** | 0.267 |
| Hym | 5.09 | 1.01 | 3.81 | 1.03 | 3.76 | 0.92 | **<0.001** | **<0.001** | 0.973 |
| Def1 | 5.11 | 0.9 | 3.9 | 0.94 | 3.66 | 0.7 | **<0.001** | **<0.001** | 0.503 |
| Vg | 4.41 | 0.55 | 4.32 | 0.64 | 4.1 | 0.61 | 0.803 | 0.112 | 0.345 |
| Nkd | 4.93 | 1.25 | 3.93 | 1.06 | 3.81 | 1.08 | **0.004** | **0.001** | 0.908 |

**Table S3.** Results of mixed linear models showing effects of the management systems, year, and management system by year interaction on the different response variables. Farm was included as a random effect as well as year number to account for repeated measures (see methods section for model syntax). Denominator degrees of freedom (ddf) were determined using Kenward-Roger approximation. P values <0.05 are shown in bold.

|  | **Management** | | | | **Year** | | | | **Management * Year** | | | |
| --- | --- | --- | --- | --- | --- | --- | --- | --- | --- | --- | --- | --- |
| **Variable** | **F** | **ndf** | **ddf** | **P** | **F** | **ndf** | **ddf** | **P** | **F** | **ndf** | **ddf** | **P** |
| Survival | 30.11 | 2 | 142 | **<0.001** | 1.5 | 2 | 97 | 0.228 | 0.76 | 4 | 145 | 0.554 |
| Honey production | 0.45 | 2 | 520 | 0.637 | 22.7 | 2 | 37 | **<0.001** | 2.9 | 4 | 520 | **0.022** |
| *Varroa* mites | 6.29 | 2 | 144 | **0.002** | 13.09 | 2 | 116 | **<0.001** | 4.97 | 4 | 146 | **0.001** |
| *V. ceranae* | 0.27 | 2 | 142 | 0.761 | 3.78 | 2 | 35 | **0.033** | 0.25 | 4 | 143 | 0.912 |
| DWV | 7.84 | 2 | 142 | **0.001** | 0.09 | 2 | 27 | 0.917 | 0.21 | 4 | 142 | 0.933 |
| Hym | 2.24 | 2 | 143 | 0.11 | 14.83 | 2 | 88 | **<0.001** | 1.23 | 4 | 144 | 0.3 |
| Def1 | 4.5 | 2 | 142 | **0.013** | 5.48 | 2 | 45 | **0.007** | 0.56 | 4 | 143 | 0.695 |
| Vg | 1.71 | 2 | 143 | 0.185 | 13.96 | 2 | 63 | **<0.001** | 0.24 | 4 | 144 | 0.916 |
| Nkd | 3.93 | 2 | 143 | **0.022** | 119.75 | 2 | 79 | **<0.001** | 1.45 | 4 | 146 | 0.222 |

**Appendix 1. Expanded Methods**

**Management Details**

Table 1. Details of the three management systems. *Mite treatments were applied to all of the colonies in the group in the specific location when one or more colonies reached a mite population over 1 mite per 100 bees (1%) during the summer. In the fall, all colonies in a given system were treated regardless of their mite loads. In year 3, fall treatment type for the organic management system was dependent on previous treatments, to ensure chemical rotation for integrated pest management.

| **Conventional** | **Organic** | **Chemical-Free** |
| --- | --- | --- |
| Equipment | | |
| 5.4 mm plastic foundation | 5.4 mm plastic foundation | 4.9 mm plastic foundation |
| All standard worker comb | 10% drone comb | 10% freeform comb |
| Screened bottom board | Solid bottom board | Solid bottom board |
| Queen excluder used | No queen excluder | No queen excluder |
| Smooth boxes | Smooth boxes | Roughened boxes |
| Wooden wintering cover | Wooden wintering cover | Cotton duck cloth cover |
| Feed | | |
| ProSweet, as needed | ProSweet, as needed | ProSweet, as needed |
| Candy board for winter | Dry sucrose for winter | None needed |
| No pollen patty | ⅓ pollen patty in Mar. | No pollen patty |
| Treatments | | |
| Year 1 | | |
| Screened bottom board | Drone brood removal | Small cell comb |
| OA dribble within 1 week | OA dribble within 1 week | None |
| Amitraz  in mid-Aug.  (ApiVar, 4 strips for 42 days) | Formic acid (Formic Pro)  in mid-Aug.  (1 pad x 2 @ 10 days) | None |
| OA vapor in Dec. | OA vapor in Dec. | None |
| Years 2 and 3 | | |
| OA vapor in Mar. | No Mar. treatment | None |
| *OA vapor Apr-June  (1g crystals x 3 @ 7 days) | *OA vapor Apr-June  (1g crystals x 3 @ 7 days) | None |
| *Formic acid Jul.-Aug.  (Formic Pro 2 pads) | *Formic acid Jul.-Aug.  (Formic Pro 2 pads) | None |
| Amitraz  in mid-Aug.  (ApiVar, 4 strips for 42 days) | ^t^Thymol or formic acid (as above) in mid-Aug.  (Apiguard, 50 g for 2 weeks, then 50 g for 4 weeks) | None |
| OA vapor in Dec. | OA vapor in Dec. | None |

*Colonies were treated only if they exceeded the 1% mite infestation threshold

^t^Treatments in the organic management system were dependent on previous treatments.

In spring of 2018, we fed all colonies ProSweet (Mann Lake Ltd.) every two weeks for the first six weeks after package installation. Thereafter, we fed them each fall, only as needed, to a minimum weight of 60 lb of stored food (120 lb total hive weight). We assessed the colonies every two weeks throughout the 2018 and 2019 beekeeping seasons, and every three weeks due to pandemic restrictions, during the 2020 beekeeping season. At each visit, we determined queen status, watched for diseases and pests, and added or removed boxes, as needed. Once each month, we measured the population of parasitic *Varroa* mites using an alcohol wash of approximately 300 bees (standard procedure). If a single colony in a group in an apiary in the conventional or organic management system tested above a threshold of 1 mite per 100 bees (1%), all of the colonies in that system in that apiary were treated using either oxalic or formic acid, depending on the season (see Table 1).

We monitored each colony for signs of swarm initiation (2019 and 2020). If any sign of swarming was seen (queen cells at any stage), the colony was split into two colonies. The new colonies (splits) were used to replace winter losses, so that the colony density remained at 12 per apiary when possible. If too many splits were made, the extras were removed from the area. We managed splits in the same way as continuous colonies, continuing the management system of the parent colony. However, we do not report on these colonies in this paper.

In mid-August each year, all colonies in the conventional and organic management systems were treated for mites, regardless of the mite population size. Colonies in the conventional management system were treated each fall with a full dose (4 strips) of ApiVar (amitraz) for 42 days, when strips were removed. Colonies in the organic management system were treated using an integrated pest management strategy. In 2018, the colonies were all treated with FormicPro (formic acid) by adding one pad to the hive for 10 days followed by a second pad for an additional 10 days. In 2019, colonies that had not been treated during the summer received FormicPro pads using two pads per hive, while colonies that had already been treated over the summer with formic acid because they reached the threshold were treated with ApiGuard (thymol) by adding 50 g of gel that remained for 14 days followed by an additional 50 g of gel that remained in place for 28 days. In 2020, summer mite levels did not lead to treatments, so all colonies were treated with Formic Pro using 2 pads. *Varroa* mite levels were reassessed after treatment, in early October. In 2018 and 2019, the mite levels were sufficiently low. In 2020, however, the mite levels at 5 of the 6 PA farms were well over the 1% threshold in both the conventional and organic management systems. Therefore, these colonies were treated with oxalic acid three times at 7-day intervals. The first of the three oxalic acid treatments was oxalic acid dribble using 1:1 syrup with 35g oxalic acid crystals per liter at a rate of 50 ml per hive. The second and third treatments were via sublimation at a rate of 1 g oxalic acid crystals per hive using a ProVap vaporizer.

***Varroa* mite quantifications**

For *Varroa* quantification, we used alcohol wash of approximately 300 bees every month of the study We shook worker bees from a frame containing an abundance of 4th and 5th instar larvae, then scooped ½ cup of bees into a jar containing alcohol. We shook the jar for 60 seconds, then drained the alcohol through a fine sieve while vigorously shaking the jar. We counted the number of mites that were removed from the bees during this alcohol wash procedure to estimate the infestation level of the mites, as is standard procedure.

***Vairimorpha ceranae* and *Vairimorpha apis* quantification**

Workers were sampled from a frame with an abundance of 4th and 5th instar larvae and stored in 75% ethanol. From each colony at each sampling period, DNA was extracted from 30 worker abdomens in pools of five abdomen for a total of six composite samples per colony per sampling period. DNA was extracted using a Bender Buffer lysis and phenol:chloroform extraction as previously described (Traver et al. 2009). From each colony at each sampling period, DNA was extracted from 30 worker abdomens in pools of five abdomens for a total of six composite samples per colony per sampling period. DNA was quantified and qPCR was performed with species-specific primers to determine whether *Vairimorpha ceranae and Vairimorpha apis* (formerly *Nosema ceranae* and *Nosema apis*; Tokarev et al. 2020). Standard curve quantitation was used to determine pathogen levels (Traver and Fell 2011).

**Virus and gene expression quantification**

Samples of in-hive worker bees were collected in October each year. We shook bees from frames with an abundance of 4th and 5th instar larvae into sampling bins and then collected bees in 50 mL conical tubes. We transported the samples from the field to the lab on dry ice until stored at - 80°C.

For RNA extraction, we removed the abdomens of 30 bees per colony. All dissections were performed in sterilized petri dishes. One sample per colony was created by pooling 30 abdomens from a colony in a plastic bag with 7 mL of Chaos buffer (4.5 M Guanidine thiocyanate, N-Lauroylsarcosine sodium, EDTA, 0.1 M beta-mercaptoethanol). Abdomens were crushed with a rolling pin for 30 seconds, and then a 300 ulL aliquot of buffer was transferred to a 1.7 mL centrifuge tube. An additional 100 uL of Chaos buffer was added to each sample and centrifuged for 3 minutes at 12000 x g (11.4 rpm). 200 μuL of supernatant was transferred to a new centrifuge tube and 200 uL of 95-100% ethanol was added to the tube. RNA was extracted from this homogenate using the Zymo Quick-RNA Microprep Kit (Catalog number R1050) according to the manufacturer’s protocol and eluted into 30 µl DNase/RNase-Free Water. Quality and quantity of the RNA was assessed using a SpectraMax iD3 Multi-Mode Microplate Reader (Molecular Devices, San Jose, CA).

*Reverse transcription and qPCR*

We quantified the viral load of the two pathogens and the expression of the four genes through quantitative reverse-transcription PCR (qRT-PCR) using previously-developed primer sequences (Table S1). Before qRT-PCR could be conducted, RNA extractions from each colony were used as templates to generate complementary DNA. cDNA was synthesized from 2 µg of RNA using random primers and MultiScribe RT, following the manufacturer’s protocol (Applied Biosystems, Foster City, CA). qRT-PCR reactions were carried out in 384 well plates using a QuantStudio 5 Real-Time PCR System (Applied Biosystems). Each well contained 5 µl of qPCR Master Mix, 0.25 µl of each of the forward and reverse primers (10 µM), 2.5 µl nuclease-free H 2 O, and 2 µl cDNA template. Reactions were carried out under the following conditions: 60s at 95°C for initial denaturation, then 40 cycles of 15s at 95°C for denaturation, and 30s at 60°C for annealing, extension, and data collection followed by a melting curve analysis of 15s at 95°C, 60s at 60°C, and 1s at 95°C to determine the specificity of amplification products. All reactions were run in triplicate and each plate included negative controls of nuclease-free water for each set of primers. Elongation factor 1-alpha (ef1-alpha) was determined to be a suitable reference gene due to its similar level of expression in all samples, and was used as the reference gene for these experiments.

The Ct value for each sample was determined by taking the mean of the three technical replicates. We subtracted the Ct value of the reference gene from the Ct value of target genes to generate ΔCt values for each sample.
